# Deciphering the tissue-specific functional effect of Alzheimer risk SNPs with deep genome annotation

**DOI:** 10.21203/rs.3.rs-3871665/v1

**Published:** 2024-02-08

**Authors:** Pradeep Varathan Pugalenthi, Bing He, Linhui Xie, Kwangsik Nho, Andrew J Saykin, Jingwen Yan

**Affiliations:** 1* Department of BioHealth Informatics, Indiana University-Purdue University Indianapolis, 420 University Blvd, Indianapolis, 46202, Indiana, United States.; 2 Department of Electrical and Computer Engineering, Indiana University-Purdue University Indianapolis, 420 University Blvd, Indianapolis, 46202, Indiana, United States.; 3 Department of Radiology and Imaging Sciences, Indiana University School of Medicine, 550 University Blvd, Indianapolis, 46202, Indiana, United States.

**Keywords:** Alzheimer’s disease, GWAS annotation, chromatin feature

## Abstract

Alzheimer’s disease (AD) is a highly heritable brain dementia, along with substantial failure of cognitive function. Large-scale genome-wide association studies (GWASs) have led to a significant set of SNPs associated with AD and related traits. GWAS hits usually emerge as clusters where a lead SNP with the highest significance is surrounded by other less significant neighboring SNPs. Although functionality is not guaranteed even with the strongest associations in GWASs, lead SNPs have historically been the focus of the field, with the remaining associations inferred to be redundant. Recent deep genome annotation tools enable the prediction of function from a segment of a DNA sequence with significantly improved precision, which allows in-silico mutagenesis to interrogate the functional effect of SNP alleles. In this project, we explored the impact of top AD GWAS hits on chromatin functions and whether it will be altered by the genetic context (i.e., alleles of neighboring SNPs). Our results showed that highly correlated SNPs in the same LD block could have distinct impacts on downstream functions. Although some GWAS lead SNPs showed dominant functional effects regardless of the neighborhood SNP alleles, several other SNPs did exhibit enhanced loss or gain of function under certain genetic contexts, suggesting potential additional information hidden in the LD blocks.

## 1 Introduction

Alzheimer’s disease (AD) is the most common form of brain dementia, associated with substantial failure of organs and mental issues. Nearly 10% of the U.S. population older than 65 years have been accounted for AD with recent cases projecting to 13.8 million by 2060 [1]. The heritability of AD is estimated to be between 60% and 80% [2]. Therefore, much work has been done in genetic association studies seeking to determine the genetic architecture of AD since the early 1990s, followed by several large-scale genome-wide association studies (GWASs) and meta-analyses [3–5]. It is expected that these increasing findings will better delineate the pathways underlying disease. However, there is a large gap between estimated heritability and that explained by existing GWAS findings [6, 7].

GWAS hits usually emerge as clusters where a lead SNP with the highest significance is surrounded by other less significant neighboring SNPs. This observation of hits in clusters aligns with the model of “haplotype blocks.” That is, genomic regions are inherited together as sets (i.e., haplotype blocks) and nearby variants within the blocks can be highly correlated, known as linkage disequilibrium (LD) [8–10]. Although functionality is not guaranteed even with the strongest associations detected in GWASs, lead SNPs have been historically the focus of the field, treating the remaining associations as redundant [11]. In polygenic risk analysis where GWAS summary statistics are used to estimate the personal genetic risk of AD, the risk effect of neighboring SNPs is commonly excluded through pruning or clumping [12, 13]. Lead SNPs have also been widely used to assist with drug discovery since drug targets with genetic evidence of disease association are more likely to succeed [14]. Yet, lead SNPs identified from GWASs have not been consistent but rather nearby the same neighborhood [15]. The susceptibility locus in AD, reported as the nearest genes to lead SNPs are sometimes different even for the same SNP [15]. Taken together, information harbored in the neighborhood of lead SNPs may not necessarily be redundant. Focusing only on the lead SNPs will likely limit our understanding of genetic factors in AD [11].

Recently, deep learning models have shown considerable promise in predicting the function of DNA sequence segment, such as transcription factor binding sites (TFBS) and histone marks [16, 17]. These models attained high accuracy in predicting the underlying chromatin marks in a tissue-specific manner [18]. Through in-silico mutagenesis, one can also examine how each individual allele affects the predicted function of the input DNA sequence. In this paper, we will utilize the recent deep learning model called Expecto to investigate the downstream functional changes associated with the top GWAS hits in AD [16, 17]. In particular, we aim to explore: 1) What are the functional changes associated with AD lead SNPs? 2) Is there any difference in functional effect between lead SNPs and others in the same LD block? and 3) Will the functional effect of AD lead SNPs will be affected by the genetic context (i.e., alleles of neighboring SNPs)?

## 2 Methods

### 2.1 GWAS candidate loci

AD risk SNPs were extracted from a large-scale genome wide association study (GWAS), the International Genomics of Alzheimer’s Project (IGAP). This study was performed with the imputed genotype of 11,480,632 single nucleotide polymorphisms (SNPs) from 21,982 Alzheimer’s disease patients and 41,944 cognitively normal controls. It is a combination of four consortia, namely, the Alzheimer Disease Genetics Consortium (ADGC), the European Alzheimer’s disease Initiative (EADI), the Cohorts for Heart and Aging Research in Genomic Epidemiology Consortium (CHARGE), and Genetic and Environmental Risk in AD Consortium Genetic and Environmental Risk in AD/Defining Genetic, and the Polygenic and Environmental Risk for Alzheimer’s Disease Consortium (GERAD/PERADES) [19]. In this study, we focused on the top 100 significant SNPs with the smallest pvalue in IGAP. In addition, neighboring SNPs located within the same linkage disequilibrium (LD) block of those top hits were also included, totalling to 238 variants. LD block information was estimated from the 1000 Genome Project uisng European population [20].

### 2.2 Deep genome annotation for allele-specific function

AD risk variants from GWASs are located predominantly in noncoding regions of the genome [21–23]. Only 7 out of the top 100 GWAS hits SNPs present in the coding region, with the rest in UTR, intronic regions, and other genetic components as detailed in Appendix B. Therefore, gene regulation is speculated to be one driving factor for Alzheimer’s disease. Recently, there has been significant progress in predicting regulatory marks from raw DNA sequences using deep learning models [16, 17, 24, 25]. More specifically, these models can generate the likelihood of functions (e.g., DNase peak or binding of a specific transcription factor) with a given DNA sequence segment. Allele-specific effect can be estimated by comparing the functional likelihood of two input sequences carrying major and minor allele respectively. For example, for DNase peak, if the likelihood generated from a sequence with major allele is much higher than that from a sequence with minor allele, this suggests a potential loss of DNase peak in minor allele carriers.

Expecto is a pretrained deep genome annotation model built on the data from the ENCODE and Roadmap Epigenomics projects [16, 17, 26, 27]. As input, a short DNA sequence centering the allele of interest is used to predict chromatin profiles, including transcription factor binding sites, histone marks, and DNase peaks across various tissues and cell types. In other words, it predicts whether any of those chromatin features exist in the input sequence. Given that the majority of GWAS findings are from noncoding regions, these chromatin profiles could reveal the critical role of gene regulation in complex diseases. Expecto is trained to predict the likelihood of 2002 chromatin features, and outperforms standard models, with median AUC ≥ 0.95 across all chromatin features.

### 2.3 Allele-specific functions without genetic context

**Fig. 1:**
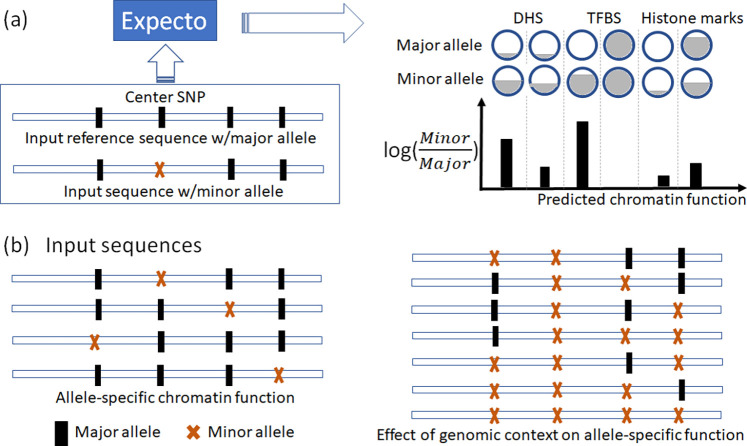
(a) Brief steps of Expecto to estimate the functional impact of the allele of interest (center SNP). The shadow inside the circle indicates the likelihood of one chromatin function given a specific input sequence. DHS: DNase I hypersensitive site, TFBS: Transcription factor binding site. (b) Input sequences used to estimate the allele-specific chromatin effect without genetic context (**left**) and with genetic context (**right**).

We first applied Expecto to evaluate the allele-specific function of candidate SNPs without considering the genetic context, i.e., all the neighboring SNPs in the input sequence set to major allele. The input for Expecto is a 2000 bp DNA sequence, centering around the SNP of interest. It was generated using the Hg38 Genome assembly as the reference genome, which was used to train the Expecto model. For each candidate SNP, two input sequences were generated: 1) one 2000 bp reference sequence directly extracted from the reference genome, 999 bp upstream and 1000 bp downstream. All the SNPs in the reference sequence were set to major alleles. 2) Another alternate sequence was generated by replacing the center SNP with minor allele. For both reference and alternate sequences, Expecto predicted the functional likelihood of all chromatin features (Fig. 1 (a)). Log odds was derived from the predicted functional likelihood, and the log odds change between reference and alternate sequences reflected the predicted functional effect of the center SNP [16].


(1) $$log(OR)=\left|log\frac{P(\mathrm{reference})}{1-P(\mathrm{reference})}-log\frac{P(\mathrm{alternate})}{1-P(\mathrm{alternate})}\right|$$

Reversed reference and alternate sequences were also examined but the predicted chromatin profiles were almost identical, so the results were not included. In addition, with a focus on Alzheimer’s disease, we manually screened all 2002 chromatin features in Expecto and included only 128 features highly relevant to brain (Appendix A). That is, these chromatin features are either from brain tissue or brain specific cell types like neuron, microglial and astrocyte. Monocyte is also included due to its close relationship with brain [28].

### 2.4 Allele-specific effect with genetic context

Next, we tested the influence of the neighboring SNPs on the allele-specific functional effect. That is, the alleles of neighboring SNPs within 2000 bp flanking region will change the functional effect of the center SNP, e.g., enhancing or weakening the binding activity of a specific transcription factor. Toward this, we generated a set of alternate sequences with in-silico mutagenesis, where the center SNP remained minor allele but neighboring SNPs selectively took minor alleles. We tested all possible combinations of major and minor alleles for neighboring SNPs, and examined whether any of the combinations would cause significant change in the functional effect of the center SNP (Fig. 1 (b)). Finally, we used the ADNI genotype dataset to validate the epistasis effects of those combinations in AD, which was downloaded from the Alzheimer’s Disease Neuroimaging Initiative (ADNI) database (adni.loni.usc.edu).

### 2.5 E value generation

To evaluate the significance of our findings, we randomly selected 1,000,000 SNPs from chromosomes 1–22 and examined their allele-specific chromatin effect on all 128 brain-related chromatin features. As such, for each chromatin feature, we obtained a distribution of log odds ratio changes. On top of that, we estimated the empirical p-values of all log odds ratios obtained using sequences around AD risk SNPs. Following [17], E-value was determined by the product of the log odds change (relative change) and the absolute change, and was formulated as follows:

(2) $$\left.\left.\left|log\frac{P(\mathrm{reference})}{1-P(\mathrm{referenc}\;)}-log\frac{P(\mathrm{alternate})}{1-P(\mathrm{alternate})}\right|*\right|\;P(\mathrm{reference})-P(\mathrm{alternate})\right|$$

## 3 Results

### 3.1 Allele-specific effect without genetic context

**Fig. 2:**
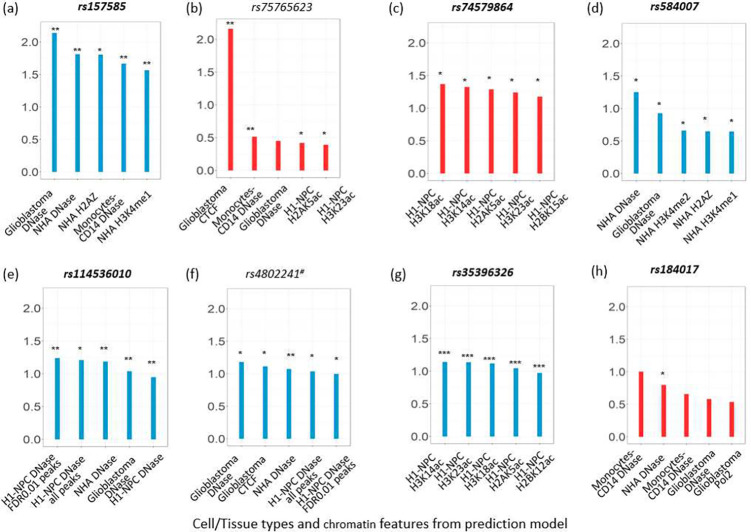
Top chromatin features affected by GWAS SNPs and their neighboring SNPs without genetic context. That is, only the center SNP in the input sequence takes minor allele. SNPs with log odds change greater than 1 in at least one chromatin features were included in the figure. Red indicates a positive log odds ratio change and blue indicates a negative log odds ratio change, suggesting gain and loss of function respectively. The variants in bold are among the top 100 AD GWAS SNPs. #: significant variants in the neighborhood of the top GWAS SNPs. *: e-value < 5e-2, **: e-value < 5e-3. ***: e-value < 1e-6. NHA: normal human astrocytes, H1-NPC: H1-derived neural progenitor cells.

After examining each individual AD risk SNP and its neighborhood SNPs, we found 8 of them with noteworthy log odds ratio changes in brain-related chromatin features. Six of those are among the top 100 AD GWAS candidate SNPs and two are in the 2000 bp neighborhood of those top SNPs, with one as GWAS significant but not the other. Among the top AD GWAS SNPs, sequences with minor allele of rs157585 was predicted to be associated with the prominent loss of function for DNase I hypersensitive sites (DHS) in glioblastoma cells, normal human astrocyte (NHA) and monocyte cells, and also histone marks in normal human astrocytes (Fig 2 (a)). Another top AD GWAS variant, rs74579864, was predicted to provide strong gain of function in acetylation of histones 2 and 3 at various positions in H1-Derived Neuronal Progenitor cells (NPC). rs35396326 is also strongly associated with acetylation of histones 2 and 3 in H1-derived Neuronal Progenitor cells, but at different positions and in a negative way. Another prominent feature predicted to have a spike in the log odds ratio was from gliobalstoma CTCF factor in rs75765623, located in the first intron of NECTIN2 gene. This variant is neither among the top GWAS SNPs nor a significant variant, but with highest log odds ratio change (e-value = 0.0458). It is located only 70bp downstream of a significant AD GWAS hit rs12462573. Yet, predicted chromatin effect associated with rs12462573 was minimal and negligible. When examined in the European population, we did not observe strong correlation between these two SNPs despite their closeness in physical location. It is therefore worth noticing that SNPs with significant p-value (i.e., lead SNPs) do not necessarily have the strongest downstream functional effect. Actual functional effect could come from less significant variants located in the neighborhood of top hits.

### 3.2 Highly correlated SNPs within LD block showed distinct allele-specific effect

**Fig. 3:**
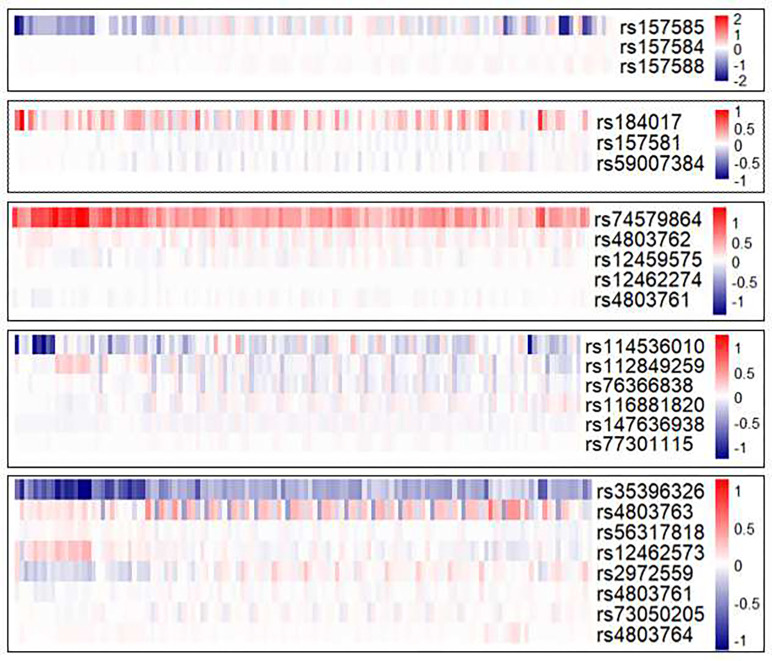
Highly correlated variants (R2 ≥ 0.8) of rs157585, rs184017, rs74579864, rs114536010 and rs35396326 from the same LD block were predicted to have different functional effects.

For those 8 SNPs predicted with significant allele-specific functional effect, we identified SNPs in the same LD block using LDLink [29] (Supplementary Fig. 1) and compared their functional effects predicted by Expecto Among 8 SNPs, 5 of them have highly correlated SNPs (corr ≥ 0.8) located in the same LD block. Shown in Fig. 3 is the comparison of predicted allele-specific functional effect across highly correlated SNP groups. Each panel is a group of correlated SNPs in the same LD block and the first row is the SNP predicted with significant allele-specific effect in the above section. Interestingly, these highly correlated variants seldom had similar predicted chromatin profiles. For example, rs74579864, rs4803761 and rs4803762 are highly correlated (*R*^2^ = 0.956 and *R*^2^ = 0.978 respectively). However, minor allele in rs74579864 was predicted to increase the likelihood of histone marks in H1-derived neuronal progenitor cultured cells, but not those of rs4803761 and rs4803762. Similarly, sequences with minor allele in rs157585 was predicted with strong negative effect in DNase hypertensive sites in monocytes CD14 and glioblastoma cells, neither of which was observed for its highly correlated neighbor rs157584 (*R*^2^ = 0.98). Taken together, our findings suggest that caution should be exercised when pruning LD block to narrow down the number of SNPs, which will likely result in a loss of information and significantly biased results.

#### Allele-specific effect with genetic context

For all the top 100 AD GWAS SNPs, we additionally examined the influence of genetic context on the allele-specific functional effect. That is, whether the alleles of neighboring SNPs within the 2000 bp window will affect the predicted functional effect of the center SNP. As shown in Fig. 1 (b) on the right, input sequences centered around each SNP were modified by varying the allele of neighboring SNPs within the 2000 bp window (i.e., major to minor allele). As such, we were able to identify 21 SNPs predicted with strong effect on chromatin features (log odds ratio change ≥ 1, *e* ≤ 0.05). Predicted chromatin effects of these input sequences were compared with allele-specific effect without considering the genetic context. Ultimately, four variants were observed with dominant effect, including rs157585, rs184017, rs114536010, and rs74579864. For each of these SNPs, input sequences carrying their minor allele were observed to have very similar functional effects on chromatin features regardless of the genetic context in the 2000 bp window. Three SNPs showed notable and significant log odds ratio changes (≥ 1, *e* ≤ 0.05), indicating the importance of the genetic context for SNP annotation.

**Fig. 4:**
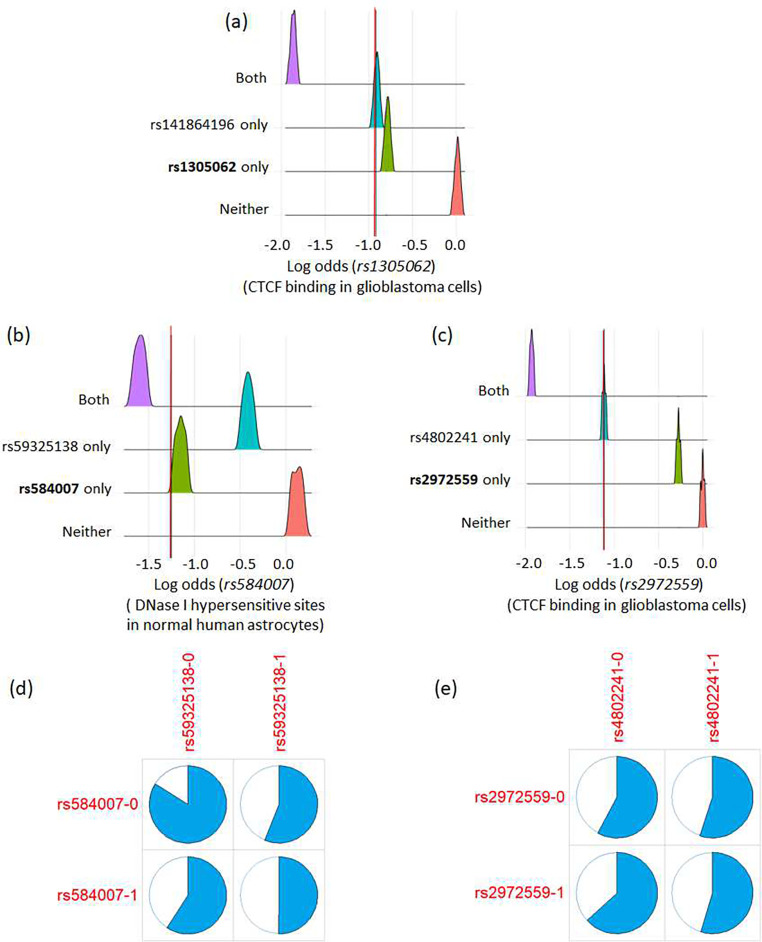
(a) Distribution of the log odds ratio change in CTCF binding in glioblastoma cells for input sequences centered around rs1305062. (b) Distribution of log odds change of DNase I hypersensitive sites in Normal Human Astrocytes for input sequences centered around rs584007. (c) Distribution of the log odds ratio changes of CTCF binding in glioblastoma cells for input sequences centered around rs2972559 (d) Proportion of subjects developing AD in the ADNI cohort grouped by rs584007/rs59325138 genotype. (e) Proportion of subjects developing AD in the ADNI cohort grouped by rs2972559/rs4802241 genotype.

We also observed notable and significant log odds ratio changes (≥ 1, *e* ≤ 0.05) for variants rs1305062, rs2972559 and rs584007, which suggests that their predicted functional effect is dependent on the allele of neighboring SNPs. For sequences carrying the minor allele in rs1305062, a significant loss of function was predicted for the CTCF binding sites in glioblastoma cells, which became even worse when the input sequence also carried the minor allele of rs141864196 (Fig. 4 (a)). A similar effect was observed for rs2972559, which in combination with rs4802241 led to a more significant loss of function in the predicted CTCF binding sites in glioblastoma cells. Interestingly, the chromatin effect observed for rs2972559 (as a top GWAS hit) alone was very weak (log odds ratio change *le* 0.5) (Fig. 4 (c)). In Fig. 4 (b), minor allele in rs584007 was predicted associated with the loss of DNase I hypersensitive sites in normal human astrocyte derived cells. This loss of function could become even more evident with the presence of minor allele in another SNP rs59325138, which had little chromatin effect as predicted by Expecto.

We further investigated the interaction effect of these three pairs of SNPs in the ADNI cohort [30]. Shown in Fig. 4 (d) and (e) are the proportion of subjects that ultimately developed AD in each genotype group (0 for no minor allele and 1 for presence of minor allele). rs141864196 was not reported due to its missing genotype in the ADNI. For the first pair of SNPs rs584007 (GWAS p = 1.056e-82, beta=−0.37) and rs59325138 (GWAS p = 6.945e-89, beta = −0.38), ratio of subjects developing AD decreases with the presence of minor allele, indicating their potential protective effect. Both SNPs are located within the LD block of lead SNP rs1081105 (i.e., top 100 GWAS hits). Combined with their predicted chromatin effect, it is speculated that deactivated DNase hypersensitive site in astrocyte cells may have a protective role in AD development. When tested in PLINK using genotype data, these two variants also showed significant epistasis effect (*p* < 1*e* − 4). For the second pair of SNPs, only rs2972559 is a significant AD risk SNP in GWASs but not rs4802241. Despite no significant epistasis effect detected in PLINK, we observed that presence of minor allele in rs4802241 to some extent mitigates the risk of developing AD introduced by the minor allele in rs2972559 (Fig. 4 (e)). Taken together, co-presence of minor alleles in rs4802241 and rs2972559 are likely associated with decreased CTCF binding in glioblastoma cells and reduced risk of AD, but their connections are yet to be further investigated.

## 4 Discussion

This study investigated the chromatin effect of sequences surrounding AD risk SNPs by leveraging deep genome annotation tools. Among the top predicted histone marks, the most significant log odds ratio changes were primarily observed in acetylation of histone H3, like H3K18ac and H3K23ac, all associated with GWAS SNP rs35396326 (beta = 0.38, p=1.35e-86 in GWAS). Acetylation levels of histones H3 and H4 have been previously reported to be overall lower in postmortem AD brains than in control brains. Among those, H3K18ac and H3K23ac were further validated as the most significantly hypoacetylated histone marks, along with H3K9ac, H3K27ac and H4K16ac [31]. In line with that, elevated levels of H3K14ac within the calpastatin promoter region was observed together with significantly decreased neuronal toxicity in neuroblastoma cells that underwent treatment to inhibit calcium-induced neuronal cell death [32]. While calcium-induced neuronal cell death is found strongly associated with the pathophysiology of AD, these evidence together suggests a potential neuroprotective role of histone acetylation. Our results provide extra support for the hypothesis that decrease in histone acetylation is associated with the minor allele of the AD risk SNP rs35396326 with positive beta coefficient (*β* = 0.38*,p* = 1.35*e* − 86 in GWASs). In other words, our findings suggest that minor allele of rs35396326 is associated with greater risk of developing AD and greater likelihood of decreased histone acetylation.

Another group of chromatin features predicted to be strong associates with the top AD GWAS hits are DNases in normal human astrocytes and monocytes. The most significant log odds ratio change in DNase activity came from *rs157585* and was specific to monocytes, astroytes and glioblastoma cells. In a DNase I footprinting analysis, mutations inside two DNAse hypersensitivite sites within recombinant *AP-2* were found associated with the regulation of the apoE promoter region, thereby implicating their role in the pathogenesis of AD [33]. Specifically, multiple DNase-I hypersenstivie sites were reported to be significantly associated to AD risk transcriptional factors in monocytes and macrophages [34]. The role of glial cells such as microglia, monocytes and astrocytes in neuroinflamation and AD have been widely studied, wherein the A*β-*activated glial cells produce cytokines and chemokines which in turn activate pathways leading to demyelination, oxidative stress and eventually cell death [35]. Although DNase I has been recently speculated to be a potential therapeutic intervention for AD, cell-type specific DNase I activity is overall under explored in AD [36, 37].

Another crucial finding of this investigation is that SNPs in the same LD block with extremely high correlation (≥ 0.9) were predicted to have very distinct effect on chromatin functions, and 2) variants that are not significant but in the neighborhood of GWAS hits could still have an impact on the downstream function (rs75762623 in Fig. 2 (b)). These results provided further proof that treating LD-block as redundant information and having one variant to represente the entire LD block could possibly bias the functional annotation of GWAS findings and our interpretation of disease mechanism.

In addition, we also observed a significant genetic context effect on the predicted functional effect of risk alleles. Among the top 100 AD GWAS SNPs, co-presence of minor alleles in two sets of neighboring SNPs were predicted with greater loss of function in CTCF binding activities in glioblastoma cells and DNase hypersentivitity sites in astrocytes. This provides evidence to support the importance of the genetic context surrounding GWAS hits, which should not be simply treated as redundant information. Similar findings have only been recently reported for other diseases such as Brugada syndrome [11]. While GWAS findings have been increasingly leveraged for many important downstream applications such as polygenic risk estimation and discovery of drug targets, caution should be exercised when utilizing the GWAS findings, especially considering the limited replicability of polygenic risk scores and failure of many clinical trials.

## 5 Conclusion

Taken together, our results suggest the need for the reanalysis of published AD GWAS data and reconsideration of future application plans for GWAS findings. This work has several limitations that merit further consideration. First, given that a long input sequence and large number of variants could lead to exponentially high number of genotype combinations as genetic context, we constrained this project to top 100 AD GWAS SNPs, which are mostly located around the APOE region and employed Expecto with 2000 bp input sequence. In addition, we examined all possible combinations of minor alleles across the SNPs within 2000 bp window, some of which may not exist. These limitations could be addressed with further haplotype estimation from phased genotype in large cohorts like UKBiobank or ADNI. Overall, this study provided a new perspective of interpreting the GWAS findings and new evidence to support the non-redundancy hypothesis of neighborhood variants surrounding GWAS hits. More in-depth work warrants further effort to investigate the functional effect of GWAS hits as clusters.

## Figures and Tables

**Fig. 5:**
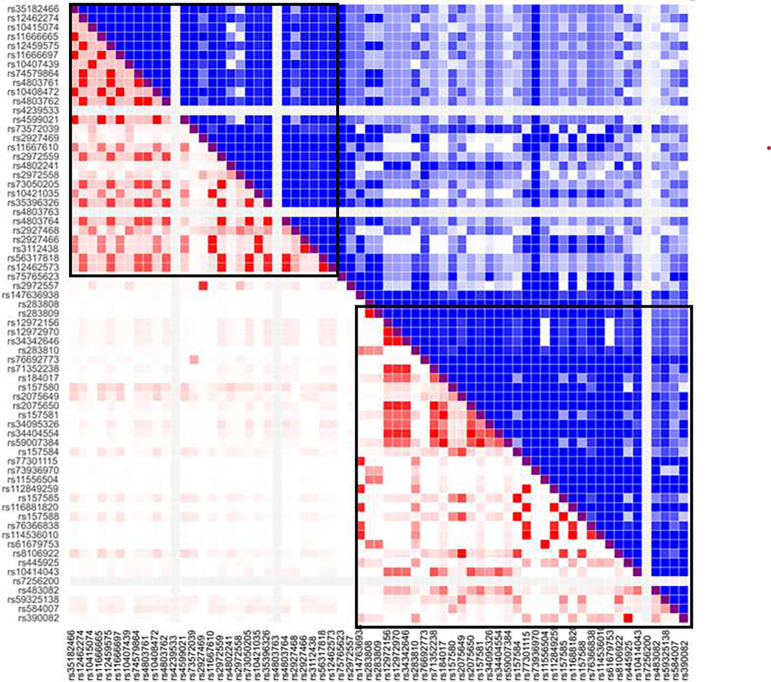
LD block of 8 SNPs that showed significant effect on chromatin features when genetic context was not considered.

## Appendix A Top AD GWAS SNPs

Top 100 AD GWAS SNPs included in this study are listed in Appendix_A.csv.

## Appendix B Brain related chromatin features in Expecto

Brain-related chromatin features related to brain were manually extracted from Expecto, and are listed in Appendix_B.csv
